# Factors associated with poor glycemic control and its correlation with albumin creatinine ratio among patients with type 2 diabetes mellitus at the University of Gondar Comprehensive Specialized Hospital, Northwest Ethiopia

**DOI:** 10.1371/journal.pone.0358345

**Published:** 2026-10-05

**Authors:** Wubet Tizazu Ferede, Markeshaw Tiruneh Gebremedhin, Workagegnehu Hailu, Endalkachew Gugsa, Tadesse Asmamaw Dejenie

**Affiliations:** 1 Department of Biochemistry, School of Medicine, College of Medicine and Health Sciences, University of Gondar, Gondar, Ethiopia; 2 Department of Internal Medicine, University of Gondar Specialized Hospital, Gondar, Ethiopia; 3 School of Health Sciences, Western Sydney University, Campbelltown, New South Wales, Australia; The University of the West Indies, JAMAICA

## Abstract

**Background:**

Monitoring for glycemic control and screening for microalbuminuria (MA) and timely therapeutic intervention have become the standard of diabetic care worldwide. However, there are limited studies conducted on factors of poor glycemic control in Ethiopia, and there is no research done on the correlation between poor glycemic control and albumin to creatinine ratio (ACR) among patients with type 2 diabetes mellitus (T2DM). Therefore, the aim of the study was to assess factors associated with poor glycemic control and its correlation with ACR among T2DM patients at the University of Gondar Comprehensive Specialized Referral Hospital, Northwest Ethiopia, 2023.

**Methods:**

An institution-based cross-sectional study was conducted from 01 April to 30 May 2023. This study included 200 T2DM patients using a systematic random sampling technique among T2DM patients who were followed up at the hospital. Data was entered and analyzed. Bivariate logistic regression analysis and Pearson’s correlation test were performed.

**Results:**

The prevalence of poor glycemic control was 82.5%. Being female (AOR = 3.74, 95% CI: 1.1, 12.83), BMI > 25 (AOR = 7.1, 95% CI: 1.37, 36.7), duration of diabetes (>5 years) (AOR = 4.35, 95% CI: 1.1, 16.9), and antidiabetic medication (insulin alone) (AOR = 8.39, 95% CI: 1.3, 56.9) were significantly associated with poor glycemic control. There was a significant positive correlation of HbA1c with the albumin creatinine ratio (r = 0.218, p-value = 0.002).

**Conclusions:**

This study concluded that majority of patients had poor glycemic control. Being female, being overweight, long duration of diabetes, and using insulin alone as antidiabetic medication were significantly associated with poor glycemic control. HbA1c also showed a significant correlation with the albumin creatinine ratio. These findings underscore the need for effective educational and therapeutic approaches by the healthcare professionals, with particular emphasis on controlling body weight and proper adherence with antidiabetic medications in diabetic patients**.**

## Introduction

Diabetes mellitus (DM) is a group of metabolic disorders characterized by elevated blood glucose levels caused by insufficient insulin secretion, insulin action, or both [1]. The most kind of diabetes mellitus, Type 2 diabetes mellitus (T2DM) is characterized by adequate insulin production but in adequate cell utilization leading to insulin resistance [2]. There are different risk factors for the development of T2DM, including family history, obesity, physical inactivity, race or ethnicity, hypertension, smoking, drinking alcohol, and dyslipidemia [3,4].

T2DM is a global health problem that causes multi-organ damage and is one of the leading causes of death and disability worldwide [5]. Despite the availability of several effective glucose-lowering treatments, over half of T2DM patients worldwide fail to meet their glycemic goals. Long-term medical expenses and the risk of complications from diabetes are increased as a result [6]. Numerous macro-vascular and micro-vascular problems such as neuropathy, nephropathy, retinopathy, cardiovascular diseases, amputations, and even early mortality are brought on by long term uncontrolled hyperglycemia [7]. The early clinical manifestation of diabetic nephropathy is an increase in urinary protein excretion [8]. Urinary albumin excretion can be measured using one of two methods: Spot urine albumin to creatinine ratio (ACR) and 24-hour albumin excretion rate (AER). ACR(mg/gm, or μg/mg) on spot urine provides useful information, ≤ 30(normal), 30–300 (MA-Microalbuminuria), >300 (macroalbuminuria) [9].

According to the American Diabetes Association (ADA), one of the primary strategies for managing diabetes mellitus is glucose control. The American Diabetes Association (ADA) views glycosylated hemoglobin (A1C) as the gold standard for glycemic management, with a goal of optimal blood glucose control of less than 6.5% in order to prevent complications and lower overall disease-related expenditures [10].

In Ethiopia, diabetic- related complications due to poor glycemic control are a major challenge for healthcare system [11]. Ongoing assessment of glycemic control routine screening for microalbuminuria, and prompt initiation of appropriate therapeutic measures are now widely recognized as essential components of standard diabetes care globally. However, little research has been done on the causes of poor glycemic control in Ethiopia, and none has been done on the relation between poor glycemic control and ACR in T2DM patients. Previous researches in Ethiopia used fasting blood glucose (FBG) to assess the degree of glycemic control. However, FBG did not shown an accurate glycemic control level. In our study we, used HbA1c to determine the glycemic control level, which is accurate and shows the average blood glucose level. Measuring albumin levels from a 24-h urine sample is the gold standard [12]. Although previous studies used 24-hour urine collection to detect diabetic- related nephropathy, ACR is better than 24-hour urine collection because 24-hour urine collection is time consuming and prone to sample collection error. Thus, the purpose of this study was to determine the causes of poor glycemic control and how they relate to early indicators of renal impairment in type 2 diabetes.

## Methods and materials

### Study design, settings, and participants

This research took place between April 1 and May 31, 2023, at the University of Gondar Comprehensive Specialized Referral Hospital, situated in the town of Gondar. The University of Gondar Comprehensive Specialized Referral Hospital’s diabetic clinic is located 750 km from Addis Ababa, the capital city of Ethiopia. The purpose of the study is to evaluate the factors associated with poor glycemic control and their relationship to the albumin to creatinine ratio (ACR) inpatients with type 2 diabetes who were undergoing routine follow-up. A total of 200 eligible T2DM patients who had follow-ups at the diabetic clinic of University of Gondar Comprehensive Specialized Referral Hospital during the study period were recruited for this study. Patients with known pre-existing medical conditions including cancer, type-1 diabetes mellitus, liver disease, congestive heart failure, HIV and those who are pregnant, are not included in the study.

A structured questionnaire was used to collect socio-demographic data including, age, sex, address, history, duration of diabetes and types of anti-diabetic drugs. Anthropometric data, including weight, height, body mass index (BMI), and blood pressure were assessed using suitable standardized instruments. The participants’ height and weight were recorded in accordance with established protocols. BMI was computed by taking weight in kilograms and dividing it by height in meters squared (BMI = Kg/m2). More over blood pressure readings were taken three times with a digital automatic BP monitoring device and the average readings for both apparatus for diastolic and systolic BP were used in this research.

Considering all sterile measures, 5 ml of blood was drawn after overnight fasting from each participant by skilled laboratory technicians for biochemical tests. A suitable test tube was used to collect the blood sample, which was then transferred to a dry, clean serum separatory tube. It was then centrifuged at ambient temperature (25°C) at 450 revolutions per minute (rpm).

Glycosylated hemoglobin A1C (HbA1c), fasting blood sugar (FBS), serum creatinine (Scr), and urea were determined using analyzed using a high-performance liquid chromatography method, and urine samples were collected to determine the urinary albumin to creatinine ratio using a quantitative test kit that was sold commercially. All the biochemical assays were performed using a fully automated analyzer at international clinic laboratory (ICL) national reference laboratory for clinical chemistry

The software program SPSS version 26 was used to clean and analyze the collected data once it was entered into Epi-data version 4.6. The continuous variables were expressed as mean and standard deviation, whereas the categorical variables were expressed as frequency and percentage, and were presented in the form of tables. To find the independent predictors of poor glycemic control Variables with p < 0.25 on Bivariate logistics regression analysis were added to a multivariate logistics regression analysis model. The relationships between urine ACR and HbA1c were determined using Pearson’s correlation test. A p value of less than 0.05 was regarded as statistically significant, and a 95% confidence range was employed.

### Ethical approval

This study was conducted after obtaining ethical approval from the Research, Community Services and Technology Transfer Chief Directorate Office with a reference number R/T/T/C/Eng./189/11/2022. A letter of request for cooperation was sent to the University of Gondar medical director office and the coordinator of the diabetic clinic OPD. Each participant gave their informed verbal consent following a thorough description of the study’s goals and methodology. Participants were made aware of their complete right to decline or stop taking part in the study at any time. Every piece of information gathered during the study was kept private.

## Results

### Sociodemographic characteristics of T2DM patients

A total of 200 patients with type2 DM participated in the study. Most of the participants were females (57.5%). The mean age with standard deviation of the participants was 60.1 + 10.68 years and ranged from 30 to 88 years. 45% of the patients were aged >60 years. One hundred thirty-four (67%) of the participants were married and sixty-one (30.5%) were single. One hundred and eighty (90%) of the participants were Urban residents. Seventy-six (38%) of the participants could not read and write, and forty-one (20.5%) of the participants had either a diploma or above first degree. Twenty-seven (13.5%) and 17 (8.5%) of the participants were employed in governmental and private, institution, respectively and one hundred forty-one (75%) of the participants were unemployed. Thirty -eight (19%) of the participants has been alcoholic and one hundred ninety-eight (98%) were nonsmokers (Table 1).

**Table 1:** Sociodemographic characteristics of study participants.

Variables	Category	Frequency (%)
Age (Years)	18-50	41(20.5)
	51-60	69(34.5)
	≥ 61	90(45)
Gender	Male	85(42.5)
	Female	115(57.5)
Residence	Urban	180(90)
	Rural	20(10)
Marital status	Single	61(30.5)
	Married	134(67)
	Divorced	5(2.5)
Educational level	Cannot read and write	76(38)
	No formal education but can write and read	4(2)
	Primary school (1–8)	32(16)
	High school (9–10)	15(7.5)
	Preparatory school (11–12)	32(16)
	Technical /vocational	13(6.5
	Higher education	28(14)
Occupation	Unemployment	142(71)
	Government and Private employee	45(22.5)
	Merchant	11(5.5)
	Others	2(1)
Alcoholic status	Yes	38(19)
	No	162(81)
Smoking	Never smoke	196(98)
	Former smoker	2(1)
	Currently smoking	2(1)

Others: student, farmer, daily laborer, house wife, house servant.

### Anthropometrics and clinical characteristics of the study participants

More than a third of the participants (35.5%) were overweight. The mean body mass index was 25.75 + 3.96. One hundred nine (54.5%) of the participants were on antidiabetic medications for more than 5 years. Ninety-one (45.5%). of the participants were on treatment for less than 5 years. The mean age of duration of diabetes was 7.84 + 6.23. The most commonly prescribed antidiabetic medication was metformin alone, (32.5%) followed by insulin (14%). More than a third (37%) of the participants were taking a combination of metformin and Glibenclamide, followed by a combination of insulin and metformin (16.5%). 21% of the participants had systolic blood pressure of greater than 140 mm Hg and eleven (5.5%) of the patients had diastolic blood pressure of greater than 90 mm Hg (Table 2).

**Table 2:** Anthropometrics and clinical characteristics of study participant.

Variables	Category	Frequency (%)	Mead (SD)
BMI (kg/m^2^)	Normal: < 25	100(50)	25.75 + 3.96
	Overweight:25–29.9	71(35.5)	
	Obese ≥30	29(14.5)	
Duration of DM (years)	<5 years	91(45.5)	7.84 + 6.23
	≥5 years	109(54.5)	
Antidiabetic medication	Metformin alone	65(32.5)	
	MTF + SU	74(37)	
	Insulin alone	28(14)	
	Insulin + MTF	33(16.5)	
Systolic BP (mmHg)	<140 mm Hg	158(79)	130.02 + 17.15
	>140 mm Hg	42(21)	
Diastolic BP (mmHg)	<90 mm Hg	189(94.5)	75.16 + 9.52
	>90 mm Hg	11(5.5)	

n = 200. BMI: body mass index, BP: blood pressure, DM: diabetes mellitus, mmHg: millimeter mercury, MTF: metformin, SD: standard deviation, SU: sulfonyl urea.

### Biochemical parameters of the study participants

The overall mean HbA1c level of the patients was 8.8%, only 17.5% of patients had HbA1c < 6.5% while one hundred sixty-five (82.5%) of the patients had poor glycemic control HbA1c ≥ 6.5%. The overall mean fasting blood sugar level of the patients was 171.5 + 64.15 mg/dl and one hundred fifty two (76%) of the patients had fasting blood sugar level of <70 and > 130 mg/dl. One-fourth (26%) of the patients had blood urea level of < 20.97 and > 43.01 mg/dl. Forty-one (20.5%) of the patients had serum creatinine level of <0.57 and >1.11 mg/dl. Sixty-nine (34.5%) of the patients had microalbuminuria, while twenty-eight (14%) of the patients had macroalbuminuria (Table 3).

**Table 3:** Biochemical parameters of the study participants.

Parameters	Category	Frequency (%)	Mean (SD)
HbA1c level (%)	<6.5%	35(17.5)	8.8 + 2.58
	≥6.5%	165(82.5)	
FBS (mg/dl)	<70 and> 130	152(76)	171.5 + 64.15
	70-130	48(24)	
Urea (mg/dl)	<20.97 and> 43.01	52(26)	17.2 + 15.9
	20.97-43.01	148(74)	
Serum Creatinine (mg/dl)	<0.57 and>1.11	41(20.5)	0.82 + 0.55
	0.57-1.11	159(79.5)	
Albumin to creatinine ratio (mg/g)	<30	103(51.5)	263.5 + 180.2
	30-299.9	69(34.5)	
	≥300	28(14)	

n = 200. dl: Deci litter; FBS: fasting blood sugar; HbA1c: glycated hemoglobin; mg: milligram; SD: standard deviation.

### Factors associated with poor glycemic control among patients with type 2 diabetes mellitus

In the bivariable analysis, the age, gender, level of education, body mass index, duration of T2DM since diagnosis, antidiabetic medication, fasting blood sugar, systolic blood pressure, and serum creatinine were significantly associated with poor glycemic control among T2DM patients at 0.25 level of significance. The multivariable analysis gender, body mass index, duration of diabetes and antidiabetic medication (insulin alone) had significant effects on poor glycemic control the 0.05 level of significance. The results of multivariate analysis showed that females (AOR = 3.74, 95% CI 1.1,12.83), BMI > 25 (AOR 7.1, 95% CI 1.37,36.7), duration of diabetes greater than 5 years (AOR 4.35, 95% CI 1.1, 16.9) and anti-diabetic medication (Insulin alone) (AOR 8.39, 95% CI 1.3,56.9) were all significantly associated with poor glycemic control (Table 4).

**Table 4:** Bivariate and multivariate logistic regression analyses of factors associated with poor glycemic control.

Variables	Category	Glycemic control (HbA1c)		COR(95%CI)	AOR(95%CI)	P value
		Good N(%)	Poor N(%)			
Gender	Male	24(28.2)	61(71.8)	1	1	
	Female	11(9.6)	104(90.4)	3.72(1.704-8.119)	3.74(1.1-12.83)	0.036*
Education	Cannot read and write	4(5.3)	72(94.7)	7.2(1.97-26.38)	–	
	No formal education but can write and read	0(0)	4(100)	0.99(6.46,0.12)	–	
	Primary school (1–8)	8(25)	24(75)	1.2(0.38, 3.77)		
	High school (9–10)	1(6.7)	14(91.3)	5.6(0.63,49.9)		
	Preparatory school (11–12)	9(28.1)	23(71.9)	1.02(0.33, 3.2)		
	Technical /vocational	5(38.5)	8(61.5)	0.64(0.16, 2.56)		
	Higher education	8(28.6)	20(71.4)	1	1	
BMI	Normal: < 25	31(31)	69(69)	1	1	
	Overweight:25–29.9	4(5.6)	67(94.4)	7.53(2.52-22.48)	7.1(1.37-36.7)	0.02*
	Obese ≥30	0(0)	29(100)	7.2(00,00)		
Duration of DM	<5 years	28(30.8)	63(69.2)	1	1	
	≥5 years	7(6.4)	102(93.6)	6.48(2.67-15.7)	4.35(1.1-16.9)	0.034*
Antidiabetic medication	Metformin alone	21(32.3)	44(67.7)	1	1	
	MTF + SU	6(8.1)	68(91.9)	5.4(2-14.5)	2.9(0.7-12)	0.143
	Insulin alone	3(10.7)	25(89.3)	3.98(1.08-4.7)	8.39((1.3-56.9)	0.03*
	Insulin + MTF	5(15.2)	28(84.8)	2.67(0.9, 7.9)	0.28(0.05-1.68)	
Systolic BP	<140 mm Hg	33(20.9)	125(79.1)	1		
	>140 mm Hg	2(4.8)	40(95.2)	5.28(1.21-22.99)		
FBS	<70 and> 130	20(13.2)	132 (86.8)	3(1.39-6.48)		
	70-130	15(31.2)	33(68.8)	1		
Serum creatinine	< 0.57 and 1.11	2(4.9)	39(95.1)	5.1(1.17-22.25)		
	0.57-1.11	33(20.8)	126(79.2)	1		

n = 200. AOR: adjusted odds ratio; BMI: body mass index; CI: confidence interval; COR: crud odds ratio; 1: references; *Significant at p < 0.05.

Being females were 3.74 times more likely to have poor glycemic control as compared to male diabetic patients (AOR = 3.74, 95% CI 1.1,12.83), type 2 diabetic patients with a BMI > 25 were 7.1 times more likely to have poor glycemic control as compared to patients with BMI < 25 (AOR 7.1, 95% CI 1.37,36.7). diabetic patients with greater than 5 years duration of diabetes since diagnosis were 4.35 times more likely to have poor glycemic control (AOR 4.35, 95% CI 1.1, 16.9) compared with those who had a shorter duration of diabetes (<5 years). Patients with diabetes who had received anti-diabetic medication Insulin alone were 8.39 times more likely to have poor glycemic control as compared to patients who had received other types of anti-diabetic medications (AOR 8.39, 95% CI 1.3,56.9) (Table 4).

### Correlation between HbA1c level and albumin-creatinine ratio

The Pearson correlation analysis, there was a significant positive correlation between the HbA1c level and albumin-creatinine ratio levels in the total study subjects (r = 0.218, p < 0.002). The correlation is weak positive and shows as HbA1c increases ACR also increases(Table 5).

**Table 5:** The correlation between HbA1C level and albumin to creatinine ratio.

Variable	Correlation coefficient (r)	P value
HbA1C (%)	0.218	0.002
Albumin to creatinine ratio (mg/g)		

n = 200, Pearson’s correlation test

## Discussion

Diabetes mellitus is a metabolic disease with several possible causes. In order to prevent or slow the advancement of the disease and lower the risk of acquiring complications from diabetes strict glycemic control is a crucial and primary technique in the treatment of individuals with type 2 diabetes. In this research, we assessed factors of poor glycemic control and its correlation with urinary albumin to creatinine ratio among ambulatory patients with T2DM in the University of Gondar Comprehensive specialized Hospital, Northwest Ethiopia.

In the current study, the prevalence of poor glycemic control was 82.5%. This finding consistent with the findings of other studies conducted in Ethiopia such as in Tikur Anbessa Specialized Hospital Addis Ababa in 2018 and 2022 were 68.3%, 73.8% [13], respectively and other parts of the country such as in India 91.8% [14] and Yemen 73.2% [15], this may be due to similar sociodemographic characteristics of the People in the countries especially in Addis Ababa. However, the magnitude of the poor glycemic control status in the current study was greater than previously reported in Brazil 47.34% [16], Peru 39.8% [17], Gobe Nigeria 50.1% [2] and Debre Markos and Felege Hiwot referral hospital 41.6% [18]. The higher proportion of poor glycemic control in the present study than in previous studies conducted in parts of the country could be because patients seeking advanced treatment were referred to the University of Gondar compressive Specialized Hospital. While previous mentioned studies assessed fasting blood glucose to determine the level of glycemic control our study measured glycated hemoglobin (HbA1C). The heterogeneity in the participants clinical features may be the cause of the discrepancy in the rate of glycemic control between our study and other studies. For instance glycemic control was evaluated in Brazil using fasting blood glucose. After a multivariate regression analysis, gender, body mass index, duration of diabetes more than 5 years, antidiabetic medication insulin alone was significantly associated with poor glycemic control at (P < 0.05).

The results of this research indicated that female patients were at a greater risk of having poor glycemic control compared with those of male patients. This result is consistent with other researches done in Yemen [15], West Shewa [19] and the Shanan Gibe Jimma Zone [20]. In this finding, being female increases poor glycemic control by 3.74 times as compared to males. This is because of less adherent to their lifestyle modification therapies, like regular physical activity this is related to obesity (29%) of the participants with BMI > 30 kg/m2 was found among female patients of this study. The other reason may be central obesity might increase the risk of poor glycemic control. This might be attributing that central obesity may be closely related to insulin resistance due to the accumulation of fat in the skeletal muscle. However, the result of this study is contradicted with that reported by other studies. Such as a study in Adama [21], revealed that being male was significantly associated with poor glycemic control than female diabetic patients, similarly the findings in Nigeria states that more males (36.4%) than females (25.4%) had poor glycemic control [22]. This may be due to genetic variations.

In this study, we found that the odds of participants with poor glycemic control were also increased with BMI (>25 kg/m2). This study is in agreement with other previous studies done in Nigeria [2] and Pakistan [23]. This is because a significant effect of obesity on poor glycemic control could be due to impaired insulin sensitivity and insulin secretion. Being overweight or obese increases the poor glycemic control of diabetes. Obesity reduces the number of receptors and reduces the target tissue’s sensitivity to insulin (diabetes mellitus Type 2).

When the course of type 2 diabetic outweighs the effects of hypoglycemic medications many people eventually require insulin therapy. However, in this study patients getting insulin alone therapy had a 3.98 fold increased risk of poor glycemic control compared to those receiving other antidiabetic medication regimens. This is consistent with earlier research from Moroccan [24] and Tikur Ambesa Hospital Addis Ababa [13]. The possible reason may be insulin injection alone may have no a tendency to reduce insulin resistance.

Furthermore, this research demonstrated a substantial correlation between poor glycemic management and a longer duration of diabetes. This conforms to that reported by other studies in south India [3], Nekemte [25],Indonesia [26] and Tikur Ambesa Hospital Addis Ababa [13]. A longer duration of diabetes is known to be associated with poor control, possibly because of the progressive impairment of insulin secretion with time due to β-cell failure. The other possible reason might be due to the chronic and progressive nature of diabetes patients with a longer duration of the disease may develop different comorbidities that may find it difficult to maintain good glycemic control [27]. Additionally, long duration of diabetes may cause different complications, such as, stroke, neuropathy and diabetic nephropathy.

Moreover, this study showed a statistically significant positive correlation of HbA1c with the urinary albumin to creatinine ratio in type2 diabetic patients (r = 0.218),(P < 0.002). This indicates that as the level of glycosylated hemoglobin increases, it causes increased urinary microalbumin excretion. This study is consistent with that reported in another study [28]. The possible reason is that hyperglycemia increases the glycation of proteins, which may lead to the formation and accumulation of harmful advanced glycation end products and oxidative stress, which might be related to early kidney function impairment and increased urine albumin-creatinine ratio in type 2 diabetes mellitus patients [29]. Thus, the albumin to creatinine ratio (ACR), measured from a random urine sample, is suggested to be an effective way to replace 24-hr urine collection for detecting microalbuminuria, which is an early predictor of diabetic nephropathy. This is because 24-h urine collection is time-consuming and subject to error [27]. Particularly, those aged and psychiatric with diabetes tend to collect 24-hr urine inaccurately because they have not good concentrations and normal activities.

### Strength and limitations of the study

This is the first study in Ethiopia to examine the correlation between poor glycemic control and ACR. Therefore, this will provide the baseline information for further studies in this particular public health concern area. The present study used HbA1c to assess glycemic control, which providing an objective measure of long-term glycemic status.

However, the cross-sectional design of the study may limit the ability to establish temporal or causal relationship between independent variables and the outcome variable. In addition, other relevant clinical parameters, such as lipid profiles, were not assessed because of limited reagent availability.

## Conclusions

Most people with type 2 diabetes mellitus have poor glycemic control. The study discovered factors that affect glycemic control. Gender, BMI, anti-diabetic medication (insulin alone) and duration of diabetes were found to be factors significantly affecting glycemic control. Additionally, a statistically significant positive connection between HbA1c and the albumin to creatinine ratio (ACR) was found in this investigation.

### Recommendations

We recommend to those hospital health workers that effective educational and therapeutic approaches should be focused on improving appropriate medication use, controlling body weight and optimizing glucose control particularly on females, and decisions regarding treatment escalation to Insulin can be made and put into action at the appropriate time. We also recommend that researchers conduct further research on poor glycemic control and its correlation with ACR on large sample sizes and other than cross-sectional study.

## Abbreviations

ACR: Albumin to Creatinine Ratio
ADA: American Diabetic Association
BMI: Body Mass Index
BUN: Blood Urea Nitrogen
DM: Diabetes Mellitus
FBS: Fasting Blood Sugar
HbA1C: Glycosylated Hemoglobin A1C
HDL-C: High Density Lipoprotein Cholesterol
IDF: International Diabetes Federation
LDL-C: Low-Density Lipoprotein Cholesterol
OPD: Outpatient Department
T2DM: Type 2 Diabetes Mellitus
TC: Total Cholesterol
TG: Triglyceride
UOGCSH: University of Gondar Compressive Specialized Hospital.
