# Supplementary material for: Factors associated with poor glycemic control and its correlation with albumin creatinine ratio among patients with type 2 diabetes mellitus at the University of Gondar Comprehensive Specialized Hospital, Northwest Ethiopia
Source: PLoS One. 2026 Oct 5;21(10):e0358345. doi: 10.1371/journal.pone.0358345 (PMC13637892; doi:10.1371/journal.pone.0358345)
Supplement: Supplementary file 1 [file pone.0358345.s002.pdf]

Point by point author response to Editor/ reviewer comments

Title: Factors Associated with Poor Glycemic Control and its Correlation with Albumin Creatinine Ratio Among Patients with Type 2 Diabetes Mellitus: University of Gondar Comprehensive Specialized Hospital, Northwest Ethiopia.

Manuscript ID: PONE-D-26-15321

Response to Editors comment

Thank you for your thoughtful and constructive feedback and we greatly appreciate your positive remarks and valuable suggestions for improvement.

Below is a point-by-point response to your comments:

Editor Comment 1: Make some revisions according to the attached similarity report, especially the content overlapping with the first source.

Author response: We thank the Editor for suggesting to revise the similarity of the content with other sources, we revise all the similarities throughout the document. But there are some standard words and phrases, numbers reported in the result, order of Authors and some others that we cannot change by other words or phrases that we put as it is.

Editor Comment 2: Add ORCID (Open Researcher and Contributor ID) to the manuscript.

Author response: We appreciate these insightful comments. As recommended, we add ORCID (Open Researcher and Contributor ID) to the manuscript.

Editor Comment 3: The headings at all levels of the article should be numbered in order as follows. Please re-number the headings in this article.

1. Introduction (first level)

2. XXXXX (first level)

2.1. XXXXX (second level)

2.1.1. XXXXX (third level)

2.1.2. XXXX (third level)

2.2. XXXXX (second level)

2.3. XXXXX (second level)

3. Result (first level)

4. Discussion (first level)

5. Conclusion (first level)

6. Recommendations (first level)

Author Contributions (no number)

Conflicts of Interest (no number)

Acknowledgments (no number)

References (no number)

Author response: Thank you for your thoughtful comments. According to the comment we have revised the manuscript and renumbered all headings in the required number.

Editor Comment 4: Abbreviations should be defined upon their first appearance in the main body, and a list of abbreviations should be provided

Author response: We appreciate the detailed comments provided by the Editors. Based on your comment we have included a list of abbreviations and ensured that each abbreviation is defined at its first appearance in the text.

Editor Comment 5: select the relevant contributor roles and list author contributions

Author response: We are grateful for the opportunity to improve our work based on your suggestions. We have selected the relevant contributor roles and have clearly listed the specific contributions of each author in the manuscript based on the format you attach.

Editor Comment 6: Delete the section titled 'Ethical consideration' or incorporate it into the main text**.**

Author response: We thank you for your thoughtful feedback on our manuscript**.** According to your suggestion we have removed the section entitled "Ethical considerations" and incorporated the relevant information into the main text of the manuscript.

Editor Comments 7: Include the digital object identifier (DOI) for all references, if available

Author response: We have revised the reference list and included the digital object identifier (DOI) for all references where available.

We hope that the revisions adequately address the Editors concerns. We thank the Editor for their constructive feedback and consideration.
