# Supplementary material for: Factors associated with poor glycemic control and its correlation with albumin creatinine ratio among patients with type 2 diabetes mellitus at the University of Gondar Comprehensive Specialized Hospital, Northwest Ethiopia
Source: PLoS One. 2026 Oct 5;21(10):e0358345. doi: 10.1371/journal.pone.0358345 (PMC13637892; doi:10.1371/journal.pone.0358345)
Supplement: Peer review history

# Author comment: Author response to Decision Letter 0

**DOI:** 10.1371/journal.pone.0358345.r001

15 May 2026

Attachment Submitted filename: Author response Letters.docx

# Peer review: Decision Letter 0

**DOI:** 10.1371/journal.pone.0358345.r002

**Authors:** Donovan Anthony McGrowder

4 Jun 2026

Please submit your revised manuscript by Jul 19 2026 11:59PM. If you will need more time than this to complete your revisions, please reply to this message or contact the journal office at plosone@plos.org. When you're ready to submit your revision, log on to https://www.editorialmanager.com/pone/ and select the 'Submissions Needing Revision' folder to locate your manuscript file.

- A letter that responds to each point raised by the academic editor and reviewer(s). You should upload this letter as a separate file labeled 'Response to Reviewers'.
- A marked-up copy of your manuscript that highlights changes made to the original version. You should upload this as a separate file labeled 'Revised Manuscript with Track Changes'.
- An unmarked version of your revised paper without tracked changes. You should upload this as a separate file labeled 'Manuscript'.

If you would like to make changes to your financial disclosure, please include your updated statement in your cover letter. Guidelines for resubmitting your figure files are available below the reviewer comments at the end of this letter.

If applicable, we recommend that you deposit your laboratory protocols in protocols.io to enhance the reproducibility of your results. Protocols.io assigns your protocol its own identifier (DOI) so that it can be cited independently in the future. For instructions see: https://journals.plos.org/plosone/s/submission-guidelines#loc-laboratory-protocols. Additionally, PLOS ONE offers an option for publishing peer-reviewed Lab Protocol articles, which describe protocols hosted on protocols.io. Read more information on sharing protocols at https://plos.org/protocols?utm_medium=editorial-email&utm_source=authorletters&utm_campaign=protocols.

As the corresponding author, your ORCID iD is verified in the submission system and will appear in the published article. PLOS supports the use of ORCID, and we encourage all coauthors to register for an ORCID iD and use it as well. Please encourage your coauthors to verify their ORCID iD within the submission system before final acceptance, as unverified ORCID iDs will not appear in the published article. *Only*  the individual author can complete the verification step; PLOS staff *cannot*  verify ORCID iDs on behalf of authors.

We look forward to receiving your revised manuscript.

Kind regards,

Donovan Anthony McGrowder, PhD., MA., MSc

Academic Editor

PLOS One

**Journal Requirements:**

1. When submitting your revision, we need you to address these additional requirements.

Please ensure that your manuscript meets PLOS ONE's style requirements, including those for file naming. The PLOS ONE style templates can be found at

https://journals.plos.org/plosone/s/file?id=wjVg/PLOSOne_formatting_sample_main_body.pdf and

https://journals.plos.org/plosone/s/file?id=ba62/PLOSOne_formatting_sample_title_authors_affiliations.pdf

2. In the ethics statement in the Methods, you have specified that verbal consent was obtained. Please provide additional details regarding how this consent was documented and witnessed, and state whether this was approved by the IRB.

3. In the online submission form, you indicated that [Data supporting these findings are confined within the manuscript and will be shared up on request to the corresponding author.]

All PLOS journals now require all data underlying the findings described in their manuscript to be freely available to other researchers, either a. In a public repository, b. Within the manuscript itself, or c. Uploaded as supplementary information.

This policy applies to all data except where public deposition would breach compliance with the protocol approved by your research ethics board. If your data cannot be made publicly available for ethical or legal reasons (e.g., public availability would compromise patient privacy), please explain your reasons on resubmission and your exemption request will be escalated for approval.

4. When completing the data availability statement of the submission form, you indicated that you will make your data available on acceptance. We strongly recommend all authors decide on a data sharing plan before acceptance, as the process can be lengthy and hold up publication timelines. Please note that, though access restrictions are acceptable now, your entire data will need to be made freely accessible if your manuscript is accepted for publication. This policy applies to all data except where public deposition would breach compliance with the protocol approved by your research ethics board. If you are unable to adhere to our open data policy, please kindly revise your statement to explain your reasoning and we will seek the editor's input on an exemption. Please be assured that, once you have provided your new statement, the assessment of your exemption will not hold up the peer review process.

5. Please amend either the title on the online submission form (via Edit Submission) or the title in the manuscript so that they are identical.

6. Please amend either the abstract on the online submission form (via Edit Submission) or the abstract in the manuscript so that they are identical.

7. If the reviewer comments include a recommendation to cite specific previously published works, please review and evaluate these publications to determine whether they are relevant and should be cited. There is no requirement to cite these works unless the editor has indicated otherwise.

[Note: HTML markup is below. Please do not edit.]

Reviewers' comments:

Reviewer's Responses to Questions

**Comments to the Author**

1. Is the manuscript technically sound, and do the data support the conclusions?

Reviewer #1: Yes

Reviewer #2: No

**********

2. Has the statistical analysis been performed appropriately and rigorously?

Reviewer #1: I Don't Know

Reviewer #2: No

**********

3. Have the authors made all data underlying the findings in their manuscript fully available?

The PLOS Data policy

Reviewer #1: Yes

Reviewer #2: Yes

**********

4. Is the manuscript presented in an intelligible fashion and written in standard English?

Reviewer #1: Yes

Reviewer #2: Yes

**********

**Reviewer #1:**  The association between glycemic control, ACR, and common risk factors in T2DM is well-studied internationally. However, data specific to the Ethiopian context (Northwest Ethiopia, University of Gondar) and the reported lack of prior local studies examining the correlation between poor glycemic control and ACR support this study for local education.

The cross-sectional approach precludes causal inference; consider clarifying this as a limitation and, if possible, recommend future prospective or longitudinal studies.

The single-center, relatively small (n=200) sample limits broader applicability; discussion should explicitly address this and suggest multicenter studies for wider relevance.

Key variables like diet, physical activity, healthcare access, or socioeconomic status seem unaddressed—acknowledge these potential confounders and strive to measure or adjust for them in future work.

Association of insulin-alone therapy with poor control may reflect treatment selection bias or advanced disease; clarify this point and avoid overinterpretation as a direct causal relationship.

Recommendations focus on weight control, especially in women; expand to include comprehensive diabetes management strategies (medication adherence, patient education, lifestyle interventions) for all at-risk groups.

**Reviewer #2:**  This cross-sectional study examined factors associated with poor glycemic control and the correlation between HbA1c and urinary albumin-to-creatinine ratio among 200 patients with type 2 diabetes attending the University of Gondar Comprehensive Specialized Referral Hospital in Northwest Ethiopia.

The novelty of the study appears limited, as the association between poor glycemic control and albuminuria in type 2 diabetes is already well established. The claimed novelty is mainly geographic, namely that this is the first study from Ethiopia, but this point is not sufficient to support a strong original contribution.

The correlation analysis between HbA1c and ACR is insufficient. The manuscript reports only a Pearson correlation coefficient without showing the actual data distribution. Given that ACR is typically right-skewed and that 14% of the participants had macroalbuminuria, the observed correlation may be driven by a small number of high-ACR cases.

The association between HbA1c and ACR was not adjusted for major confounders such as blood pressure, BMI, duration of diabetes, age, sex, and renal function. Although BMI and blood pressure were measured, they were not incorporated into a multivariable model with ACR or albuminuria as the outcome. Therefore, the current analysis does not establish an independent association between poor glycemic control and ACR.

**********

6. PLOS authors have the option to publish the peer review history of their article (what does this mean?). If published, this will include your full peer review and any attached files.

If you choose “no”, your identity will remain anonymous but your review may still be made public.

Do you want your identity to be public for this peer review? For information about this choice, including consent withdrawal, please see our Privacy Policy.

Reviewer #1: **Yes:**  Dr. Krishna Baradhi

Reviewer #2: No

**********

[NOTE: If reviewer comments were submitted as an attachment file, they will be attached to this email and accessible via the submission site. Please log into your account, locate the manuscript record, and check for the action link "View Attachments". If this link does not appear, there are no attachment files.]

To ensure your figures meet our technical requirements, please review our figure guidelines: https://journals.plos.org/plosone/s/figures

You may also use PLOS’s free figure tool, NAAS, to help you prepare publication quality figures: https://journals.plos.org/plosone/s/figures#loc-tools-for-figure-preparation.

NAAS will assess whether your figures meet our technical requirements by comparing each figure against our figure specifications.

# Author comment: Author response to Decision Letter 1

**DOI:** 10.1371/journal.pone.0358345.r003

19 Aug 2026

Dear Academic Editor and esteemed reviewers,

The authors are grateful for your constructive comments and continuous follow-up on our submission.

We have diligently addressed all the comments and uploaded the point-by-point response file. We kindly invite you to review the separate document.

Attachment Submitted filename: Authors Response.pdf

# Editor report: Decision Letter 1

**DOI:** 10.1371/journal.pone.0358345.r004

**Authors:** Donovan Anthony McGrowder

1 Sep 2026

Factors Associated with Poor Glycemic Control and its Correlation with Albumin Creatinine Ratio Among Patients with Type 2 Diabetes Mellitus at the University of Gondar Comprehensive Specialized Hospital, Northwest Ethiopia

PONE-D-26-15321R1

Dear Dr. Ferede,

We’re pleased to inform you that your manuscript has been judged scientifically suitable for publication and will be formally accepted for publication once it meets all outstanding technical requirements.

Within one week, you’ll receive an e-mail detailing the required amendments. When these have been addressed, you’ll receive a formal acceptance letter and your manuscript will be scheduled for publication.

An invoice will be generated when your article is formally accepted. Please note, if your institution has a publishing partnership with PLOS and your article meets the relevant criteria, all or part of your publication costs will be covered. Please make sure your user information is up-to-date by logging into Editorial Manager at Editorial Manager® and clicking the ‘Update My Information' link at the top of the page. For questions related to billing, please contact billing support.

If your institution or institutions have a press office, please notify them about your upcoming paper to help maximize its impact. If they’ll be preparing press materials, please inform our press team as soon as possible -- no later than 48 hours after receiving the formal acceptance. Your manuscript will remain under strict press embargo until 2 pm Eastern Time on the date of publication. For more information, please contact onepress@plos.org.

Kind regards,

Donovan Anthony McGrowder, PhD., MA., MSc

Academic Editor

PLOS One

# Editor report: Acceptance letter

**DOI:** 10.1371/journal.pone.0358345.r005

**Authors:** Donovan Anthony McGrowder

PONE-D-26-15321R1

PLOS One

Dear Dr. Ferede,

I'm pleased to inform you that your manuscript has been deemed suitable for publication in PLOS One. Congratulations! Your manuscript is now being handed over to our production team.

At this stage, our production department will prepare your paper for publication. This includes ensuring the following:

* All references, tables, and figures are properly cited

* All relevant supporting information is included in the manuscript submission,

* There are no issues that prevent the paper from being properly typeset

You will receive further instructions from the production team, including instructions on how to review your proof when it is ready. Please keep in mind that we are working through a large volume of accepted articles, so please give us a few days to review your paper and let you know the next and final steps.

Lastly, if your institution or institutions have a press office, please let them know about your upcoming paper now to help maximize its impact. If they'll be preparing press materials, please inform our press team within the next 48 hours. Your manuscript will remain under strict press embargo until 2 pm Eastern Time on the date of publication. For more information, please contact onepress@plos.org.

You will receive an invoice from PLOS for your publication fee after your manuscript has reached the completed accept phase. If you receive an email requesting payment before acceptance or for any other service, this may be a phishing scheme. Learn how to identify phishing emails and protect your accounts at https://explore.plos.org/phishing.

If we can help with anything else, please email us at customercare@plos.org.

Thank you for submitting your work to PLOS One and supporting open access.

Kind regards,

PLOS One Editorial Office Staff

on behalf of

Dr. Donovan Anthony McGrowder

Academic Editor

PLOS One
